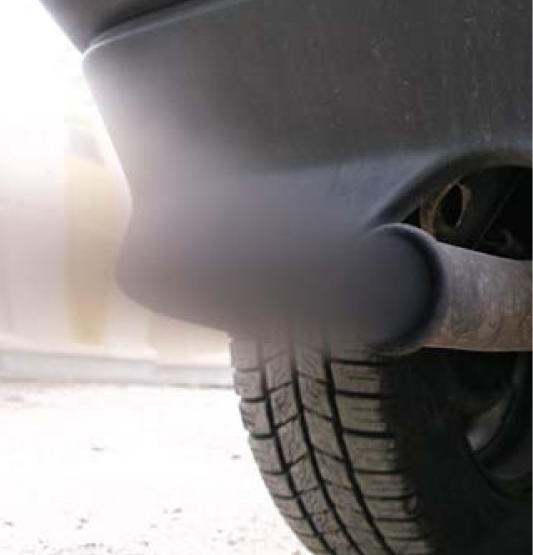# Urban Issues: A Climate for Pollution

**Published:** 2008-04

**Authors:** Adrian Burton

Carbon dioxide (CO_2_) emissions may be killing people by increasing airborne concentrations of other pollutants including ozone, particulate matter, and carcinogens such as formaldehyde and benzene, a Stanford University scientist reports in the 12 February 2008 issue of *Geophysical Research Letters*. The situation is only likely to get worse, especially in already-polluted areas, according to author Mark Jacobson, a professor of civil and environmental engineering.

CO_2_ causes temperatures to rise, which in turn increases evaporation, leading to higher water vapor content in the air. Jacobson has now confirmed the effect of these CO_2_-induced changes on ozone production using an algorithm that can quantify the reactions between hundreds of different airborne compounds over time.

He examined different scenarios involving more than 100 gases and nearly 400 inorganic and organic air pollutants. When nitrogen oxides (NO_x_; ozone precursors found in car exhaust fumes) were present at greater than 80 ppb by volume (ppbV) in the presence of organic acids, he found the ozone concentration increased by 2.8 ppbV per 1 ppth by volume water vapor. The higher the water vapor content of the air, the more readily further ozone precursors were formed.

Increasing temperature also contributed to increased ozone levels—again, the more ozone there was to start with, the more was made. A 1°C rise was associated with a 0.1-ppbV increase in ozone when the gas was present at 40 ppbV, and with a 6.7-ppbV increase when it was present at 200 ppbV. The reason: higher ozone levels are accompanied by higher levels of unstable peroxyacyl nitrate. When the latter compound breaks down as the temperature rises, it forms more NO_x_ and volatile organic compounds, thus providing further ozone precursors.

The data mass produced by the algorithm was then used in a three-dimensional climate–air pollution model known as GATOR-GCMOM to determine how CO_2_ emissions might affect ozone and other atmospheric variables over different areas of the United States. Two simulations were run, one at present CO_2_ concentrations and one at pre–Industrial Era concentrations, leaving all other variables equal.

For every 1°C rise, these emissions were associated with increases in ozone of 0.12 ppbV for the United States as a whole, but by up to 5 ppbV in heavily polluted areas such as Los Angeles. In some areas, fine particulate matter rose by an average of 0.1 μg/m^3^ per 1°C rise, whereas carcinogenic compounds experienced small increases. According to the current epidemiologic literature, says Jacobson, that could mean around 350–1,800 extra noncancer deaths per year per CO_2_-associated 1°C rise in the United States alone, and up to 39,000 deaths worldwide, with the worst hikes occurring in polluted cities. U.S. cancer deaths could rise by 20–30 per CO_2_-associated 1°C rise.

“This paper makes an interesting contribution to the literature on the less direct effects of climate change,” remarks Douglas Crawford-Brown, a professor of environmental sciences and engineering at the University of North Carolina at Chapel Hill. “While the results are even more speculative than estimates of the direct effects [such as storms and increased drought], they do point to . . . a plausible argument that these indirect effects may rival the direct effects in scale of public health concern.”

The results may help California and 19 other states in their suit against the U.S. Environmental Protection Agency (EPA) over the recent denial of a waiver for the states to set their own tailpipe emission standards, partly on the grounds that climate change does not affect them in any extraordinary way. Bart Croes, chief of the Research Division at the California Air Resources Board, says this new research shows that California, home of three of the four most polluted U.S. areas, could suffer 300 deaths per 1°C rise for every 700 in the rest of the country—a notable number given its 12% share of the population. According to the 2006 report *Our Changing Climate: Assessing the Risks to California* by the California Climate Change Center, the state can expect at least a 1.7°C rise in average temperatures by the end of this century.

Meanwhile, on 12 March 2008 the EPA signed into law a new 8-hour standard for ground-level ozone of 0.075 ppm. Under the former standard, ozone levels as high as 0.084 ppm were considered to be in compliance.

## Figures and Tables

**Figure:**